# Supplementary material for: Controlling protein activity by dynamic recruitment on a supramolecular polymer platform
Source: Nat Commun. 2018 Jan 4;9:65. doi: 10.1038/s41467-017-02559-0 (PMC5754363; doi:10.1038/s41467-017-02559-0)
Supplement: Supplementary file 1 — Supplementary Information [file 41467_2017_2559_MOESM1_ESM.pdf]

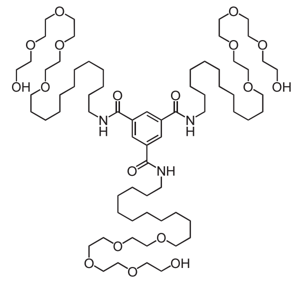

BTA-3OH

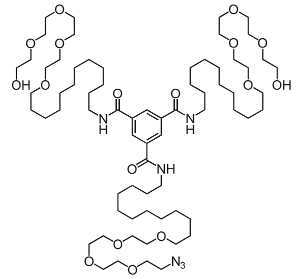

BTA-N_3

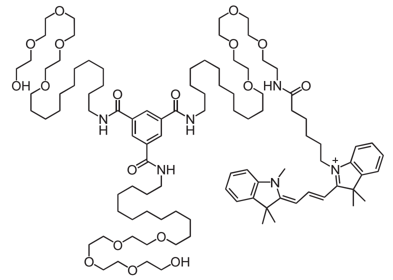

BTA-Cy3

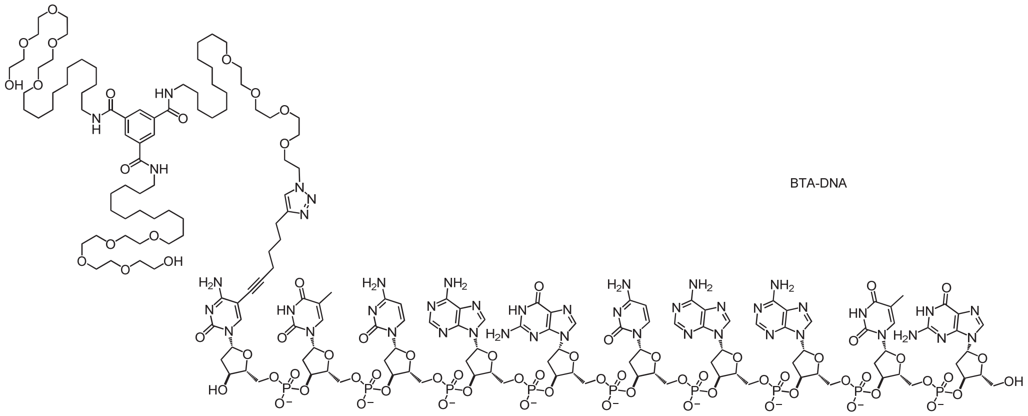

BTA-DNA

Supplementary Figure 1 | Chemical structures of BTA-3OH, BTA-N_3, BTA-Cy3 and BTA-DNA. All BTA molecules used in this work are derivatives of the water-soluble BTA developed in our group (BTA-3OH), containing an aliphatic C_{12} spacer and four ethylene glycol units per arm. BTA-DNA is obtained via azide-alkyne click conjugation between a BTA containing a terminal azide on one arm (BTA-N_3) and a DNA oligo functionalized with an alkyne.

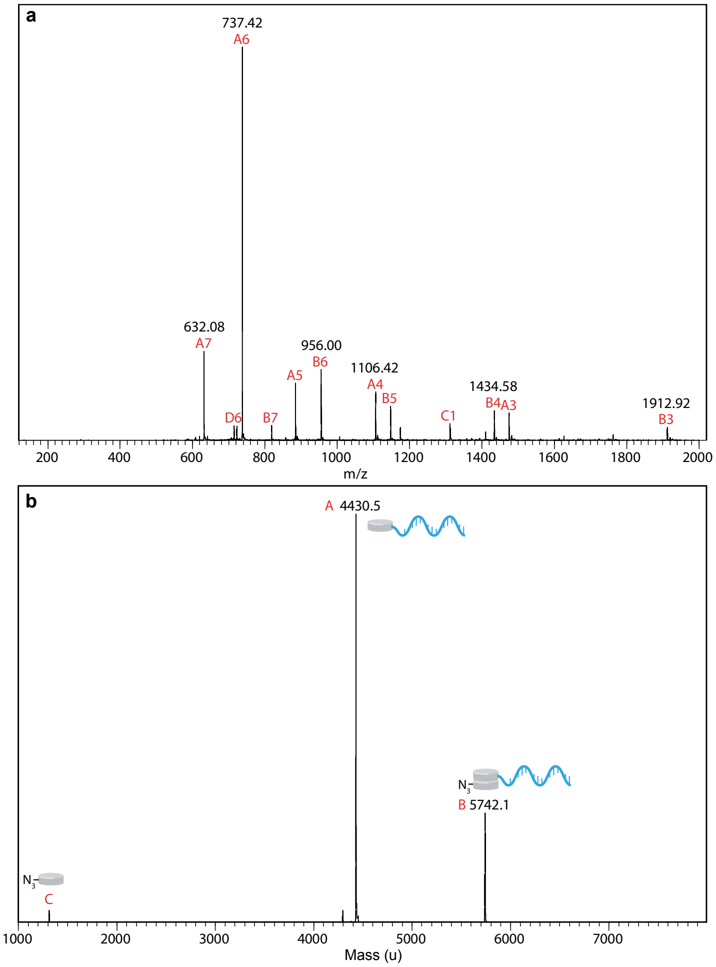

Supplementary Figure 2 | ESI-MS analysis of BTA-DNA. (**a**): Mass-to-charge spectrum. (**b**): Deconvoluted mass spectrum. Calculated masses: [BTA-N_3] = 1313.81, [BTA-DNA] = 4429.98 and the complex [BTA-DNA + BTA-N_3] = 5743.79. ESI-MS analysis of BTA-DNA shows the successful conjugation of the oligonucleotide to BTA-N_3 via a copper mediated cycloaddition reaction (peak A). A minor fraction of BTA-N_3 remains present after purification by dialysis but is not expected to have an influence on the performance of the system (peak C). Interestingly, in the ESI-MS analysis the remaining BTA-N_3 is predominantly observed in complex with BTA-DNA (peak B).

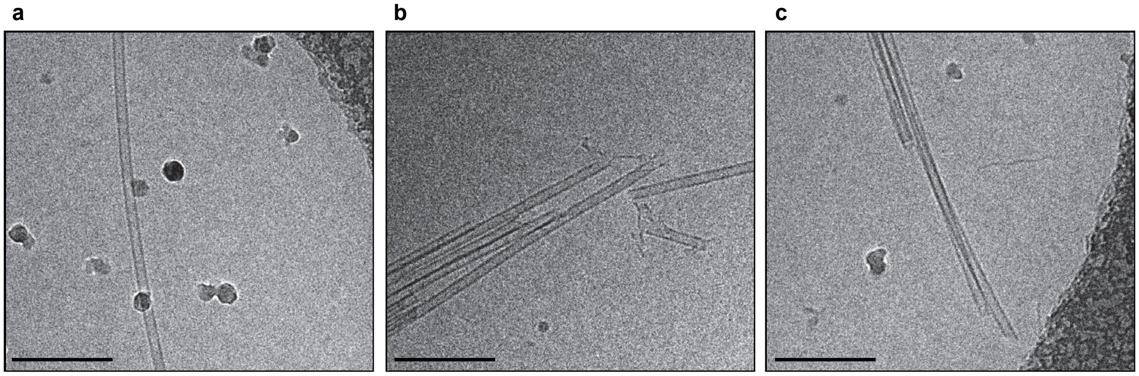

Supplementary Figure 3 | Structural characterization of DNA-decorated BTA polymers by Cryo-TEM. Images of DNA-decorated BTA polymers in PBS (50 mM sodium phosphate, 500 mM NaCl, pH 7.0) with 195 µM BTA-3OH and 5 µM BTA-DNA (2.5% BTA-DNA), 2 µM R_E and R_I. The scale bars represent 200 nm. The dark spherical objects present in the images are crystalline ice particles. (**a, b**) The images show tubular structures formed by DNA decorated BTAs. (**c**) These structures coexist with standard one-dimensional BTA fibers.

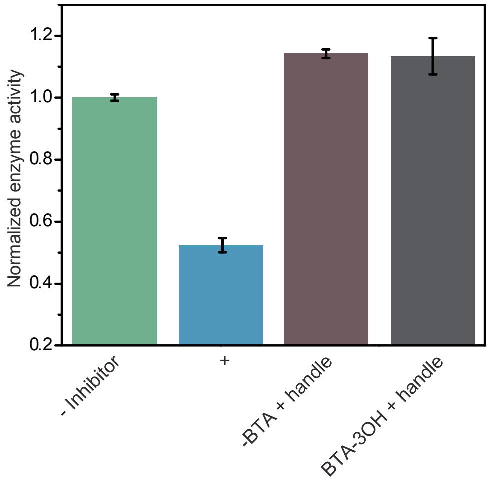

Supplementary Figure 4 | Specific BTA-templated enzyme-inhibitor complex formation. Normalized enzyme activity in the absence of BLIP (-inhibitor), the fully assembled system (+), a control lacking the BTA polymers but with a 0.5 µM 10 nt DNA strand similar to the handle on BTA-DNA (-BTA + handle) and a control with 2 µM 100% BTA-3OH and 0.5 µM of the free 10 nt DNA handle (BTA-3OH + handle). The fully assembled system (+) consisted of 25% BTA-DNA (0.5 µM BTA-DNA, 1.5 µM BTA-3OH), 20 nM R_E and R_I, 1 nM β-lactamase, 10 nM BLIP. Enzyme activities were obtained from the kinetic traces by deriving the slope of the initial increase in fluorescence. Enzyme activities were normalized to a control omitting the inhibitor protein. Error bars represent SEM of duplicate measurements.

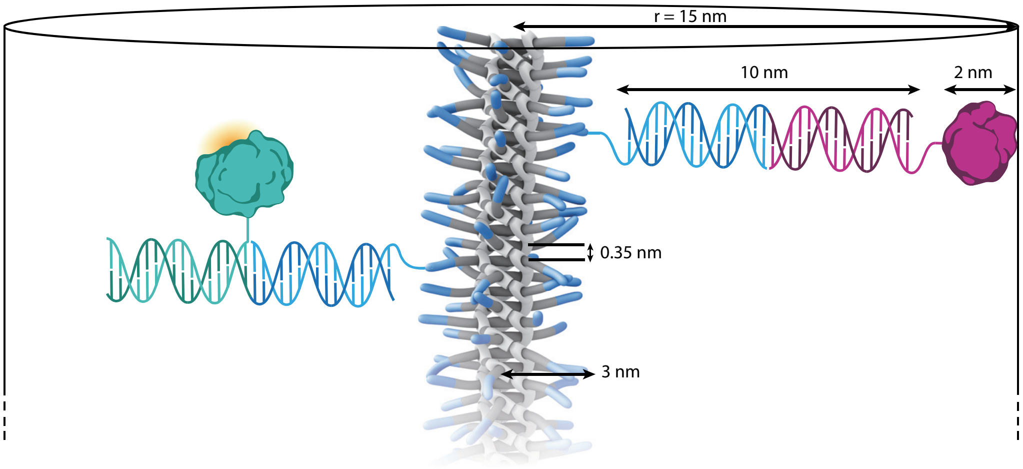

Supplementary Figure 5 | Estimation of the effective concentration of BLIP on the BTA polymers. A polymer containing 25% BTA-DNA is represented as a cylinder with a radius of 15 nm and a length determined by the amount of BTAs. The radius of the BTA polymers is determined to be 3 nm, the length of a 31 base-pair double strand DNA helix is 10 nm and the diameter of the proteins is about 2 nm.[3]

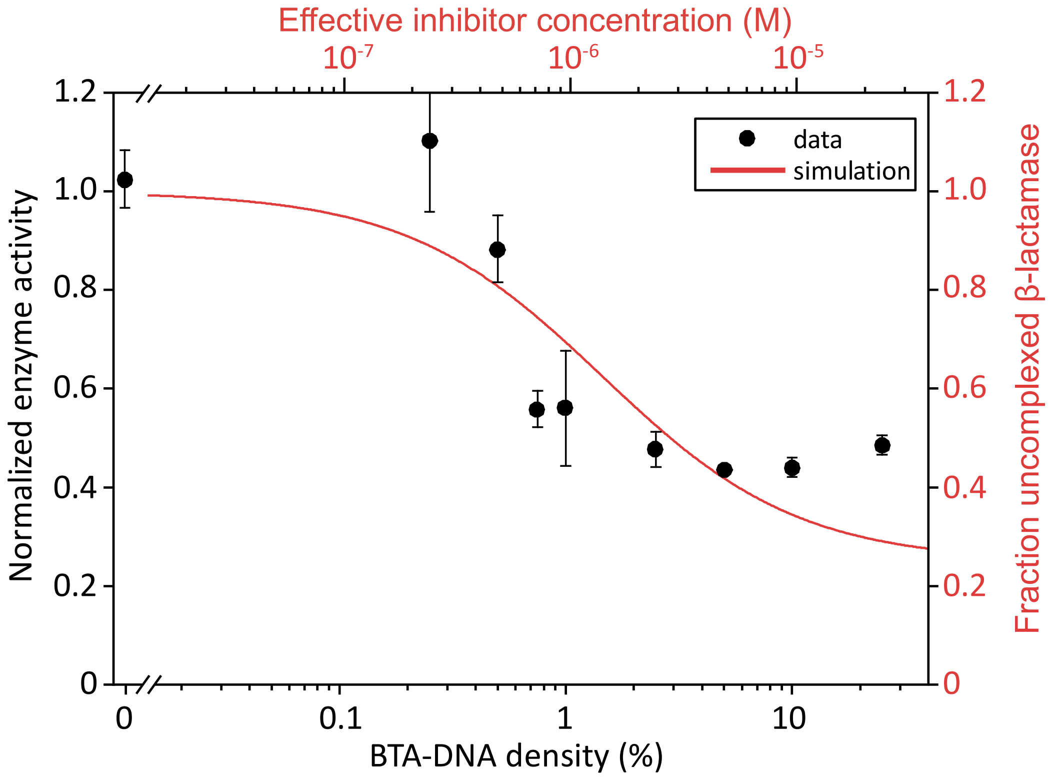

Supplementary Figure 6 | Simulation of the fraction of unbound β-lactamase as a function of effective inhibitor concentration. Assuming a β-lactamase concentration of 1 nM and an intermolecular inhibitory constant of 1.5 μM, the effective inhibitor concentrations are derived from the BTA-DNA density in the polymer based on the calculations in Supplementary note 2. The normalized enzyme activities represented by the black dots are the enzyme activities from figure 3d.

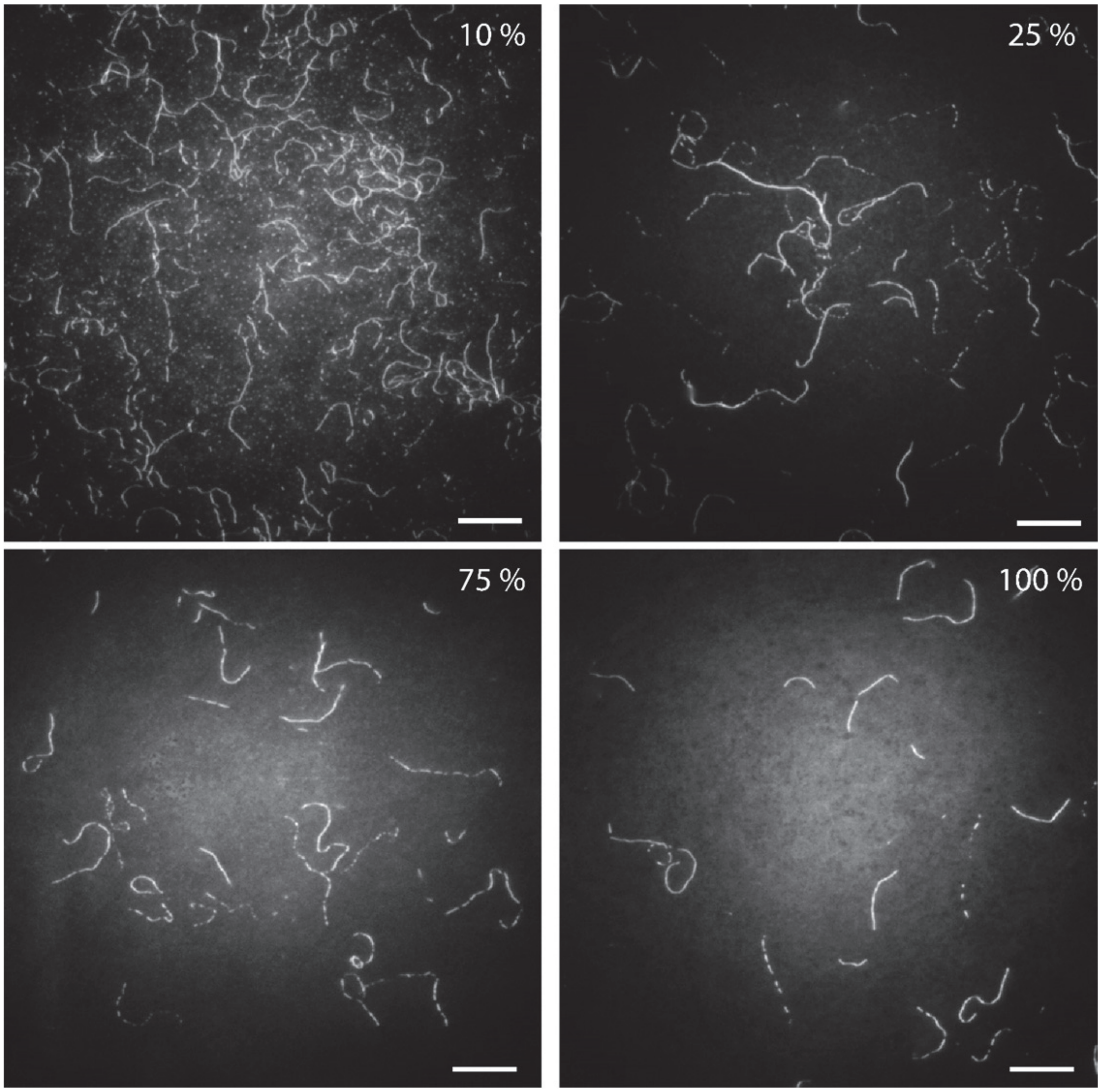

Supplementary Figure 7 | Structural characterization of DNA-decorated BTA polymers by TIRF. Images of BTA polymers containing 10, 25, 75 and 100% BTA-DNA in PBS. The polymers were stained with 5% Nile Red (relative to the total amount of BTAs). The scale bars represent 10 µm. These images show that even at extremely high percentages of BTA-DNA, micrometer long polymer are formed.

Supplementary Table 1 | The sequences and modifications of oligonucleotides used in this work.

**Strand name**	**Sequence**
Handle	5'- GTAACGACTC -3'
BTA-DNA handle	5'- GTAACGACTC-Alkyne -3'
β-Lactamase handle	5'- TGTCACCGATGAAACTGTCTA-NH_2 -3'
BLIP handle	5'- H_2N-GTGATGTAGGTGGTAGAGGAA -3'
Enzyme Recruiter strand (R_E)	5'- GAGTCGTTACTAGACAGTTTCATCGGTGACA -3'
Inhibitor Recruiter strand (R_I)	5'- GAGTCGTTACTTCCTCTACCACCTACATCAC -3'
Enzyme Recruiter strand + Toehold (R_{ET})	5'- GAGTCGTTACTAGACAGTTTCATCGGTGACATGCATCCAGA -3'
Enzyme Displacer strand (D)	5'- TCTGGATGCATGTCACCGATGAAACTGTCTAGTAACGACTC -3'

Supplementary Table 2 | Effective concentrations (C_{eff}) of BLIP on BTA polymers with varying densities of BTA-DNA.

Density of BTA-DNA	C_{eff} (μM)
0.25 %	0.258
0.5 %	0.515
0.75 %	0.773
1.0 %	1.03
2.5 %	2.58
5.0 %	5.15
10 %	10.3
25 %	25.8
50 %	51.5
75 %	77.3
100 %	103

Supplementary Note 1 | Understanding the origin of the residual enzyme activity at saturating inhibitor concentrations.

Figure 3c shows that even at saturating inhibitor concentrations a substantial amount of residual enzyme activity is observed. As the DNA-handle on the BTA monomer is relatively short, it is likely that the binding of the recruiter strands to these handles is not quantitative, hence not all enzyme is recruiter to the polymer platform. To determine the efficiency of binding of the recruiter strand to the polymer, the fraction of unbound recruiter strand was calculated as a function of total recruiter strand concentration. To this end, the dissociation constant ($K_{d,duplex}$) of the duplex formed between the handle on the BTA and the recruiter strand was calculated based on the Gibbs free energy determined with NUPACK.[1,2] Because the 3' cytosine was modified for conjugation to the BTA, we assumed that this nucleotide would not be available for DNA hybridization. Therefore, this cytosine was excluded from calculating the duplex stability, yielding $\Delta G° = -11.98$ kcal mol^{-1} and $K_{d,duplex} = 111$ nM. Using this dissociation constant, a recruiter concentration of 220 nM (20 nM R_E and 200 nM R_I) and a BTA-DNA concentration of 500 nM, it can be calculated that 24.8% of the recruiter strands, and thus 24.8% of the enzyme is not recruited to the polymer platform. This number corresponds well with the amount of residual enzyme activity observed in the presence of saturating amounts of BLIP (Figure 3c).

Supplementary Note 2 | Estimation of the effective protein concentration on the BTA polymers.

To obtain an estimate of the effective concentration of the inhibitor protein after recruitment to the BTA polymers, the accessible volume of the polymer is represented by a cylinder with a radius of 15 nm (Supplementary Figure 5). This radius is based on the diameter of the proteins (Ø = 2 nm), the radius of the BTA polymers (r = 3 nm) and length (10 nm) of the double helix formed by the DNA-handles and recruiter strands.[3] An intermolecular spacing of 0.35 nm for adjacent BTA monomers is based on previously performed simulations.[3]

If we use the experimental conditions as used for Figure 3a and b, with 10 nM BLIP, 500 nM BTA-DNA and 1500 nM BTA-3OH and take the 25% unbound BLIP into account, on average 7.5 inhibitors are recruited to 2000 BTAs. Given the intermolecular spacing of 0.35 nm for adjacent BTA monomers the volume of a cylinder with a radius of 15 nm and height of 2000 BTA monomers can be calculated using Supplementary Equation 1.

$$\pi r^2 \cdot h = \pi (15 \cdot 10^{-9})^2 \cdot 0.7 \cdot 10^{-6}\ \mathrm{m}^3 \qquad (1)$$

$$= 4.95 \cdot 10^{-22}\ \mathrm{m}^3 = 4.95 \cdot 10^{-19}\ \mathrm{L}$$

Converting the amount of BLIP molecules to moles by using Supplementary Equation 2 and dividing this over the volume of the cylinder containing the BLIP molecules in Supplementary Equation 3, yields an effective concentration of 26 µM, which is 2600-fold higher than the actual inhibitor concentration of 10 nM:

$$\frac{7.5}{N_A} = 1.275 \cdot 10^{-23}\ \text{moles} \qquad (2)$$

$$\frac{1.275 \cdot 10^{-23}}{4.95 \cdot 10^{-19}} = 2.6 \cdot 10^{-5}\ \mathrm{M} = 26\ \mu\mathrm{M} \qquad (3)$$

This 2600-fold increase in effective concentration explains the experimentally found significant increase in the apparent affinity of the protein. Supplementary Table 2 shows that the calculated effective inhibitor concentration decreases below the dissociation constant of the enzyme-inhibitor complex when the increase in total BTA results in a percentage of BTA-DNA of 1% or lower. This closely resembles the observed increase in enzyme activity when polymers were used with densities of BTA-DNA below 0.75% (Figure 3d).

Taking an enzyme concentration of 1 nM with the known inhibitory constant (K_i) of 1.5 µM between β-lactamaseE104D and BLIP, the fraction of enzyme that is not complexed with an inhibitor protein can be simulated as a function of effective inhibitor concentration. Supplementary Figure 6 shows the simulated fraction of uncomplexed enzyme as a function of effective inhibitor concentration, where the effective inhibitor concentration was calculated based on the BTA-DNA density.

Additionally, the enzyme activities as a function of BTA-DNA density shown in figure 3a are plotted over the simulated fraction of unbound enzyme.

Supplementary Note 3 | Estimation of inter-protein distances on the supramolecular polymers.

Taking the experimental conditions as used in Figure 3a and b, with 1.5 µM BTA-3OH, 0.5 µM BTA-DNA, 1 nM β-lactamase, 10 nM BLIP and 20 nM R_E and R_I, the average distance between a single recruited enzyme and the nearest inhibitor protein can be calculated as follows: Assuming that one enzyme and 10 inhibitors are homogeneously recruited on 2000 BTAs (1 nM β-lactamase, 10 nM BLIP and 2 µM total BTA), the proteins are distributed over 2000 · 0.35 nm = 700 nm. Meaning that on average, the proteins are separated by ~63.5 nm. Taking into account that 25% of each protein is not recruited on the polymer, this spacing increases as there are 0.75 enzymes and 7.5 inhibitor proteins per 2000 BTAs. This results in an average spacing of ~85 nm between the proteins.

Supplementary References

1. Zadeh, J. N. *et al.* NUPACK: Analysis and design of nucleic acid systems. *J. Comput. Chem.* **32,** 170–173 (2011).
2. Zhang, D. Y. & Winfree, E. Control of DNA strand displacement kinetics using toehold exchange. *J. Am. Chem. Soc.* **131,** 17303–17314 (2009).
3. Baker, M. B. *et al.* Consequences of chirality on the dynamics of a water-soluble supramolecular polymer. *Nat. Commun.* **6,** 6234 (2015).
